# Deep learning with an attention mechanism for continuous biomechanical motion estimation across varied activities

**DOI:** 10.3389/fbioe.2022.1021505

**Published:** 2022-10-17

**Authors:** Guanlin Ding, Andrew Plummer, Ioannis Georgilas

**Affiliations:** Department of Mechanical Engineering, University of Bath, Bath, United Kingdom

**Keywords:** deep learning, attention mechanism, gait analysis, seamless transition, varied terrains

## Abstract

Reliable estimation of desired motion trajectories plays a crucial part in the continuous control of lower extremity assistance devices such as prostheses and orthoses. Moreover, reliable estimation methods are also required to predict hard-to-measure biomechanical quantities (e.g., joint contact moment/force) for use in sports injury science. Recognising that human locomotion is an inherently time-sequential and limb-synergetic behaviour, this study investigates models and learning algorithms for predicting the motion of a subject’s leg from the motion of complementary limbs. The novel deep learning model architectures proposed are based on the Long Short-Term Memory approach with the addition of an attention mechanism. A dataset comprising Inertial Measurement Unit signals from 21 subjects traversing varied terrains was used, including stair ascent/descent, ramp ascent/descent, stopped, level-ground walking and the transitions between these conditions. Fourier Analysis is deployed to evaluate the model robustness, in addition to assessing time-based prediction errors. The experiment on three unseen test participants suggests that the branched neural network structure is preferred to tackle the multioutput problem, and the inclusion of an attention mechanism demonstrates improved performance in terms of accuracy, robustness and network size. An experimental comparison found that 57% of the model parameters were not needed after adding attention layers meanwhile the prediction error is lower than the LSTM model without attention mechanism. The attention model has errors of 9.06% and 7.64% (normalised root mean square error) for ankle and hip acceleration prediction respectively. Also, less high-frequency noise is present in the attention model predictions. We conclude that the internal structure of the proposed deep learning model is justified, principally the benefit of using an attention mechanism. Experimental results for biomechanical motion estimation are obtained, showing greater accuracy than only with LSTM. The trained attention model can be used throughout despite transitioning between terrain types. Such a model will be useful in, for example, the control of lower-limb prostheses, instead of the need to identify and switch between different trajectory generators for different walking modes.

## 1 Introduction

The prediction of biomechanical motion is required to estimate gait cycle, assess joint malfunction, and generate motion trajectories for wearable rehabilitation devices (Rai et al., 2019; Giarmatzis et al., 2020; Lee et al., 2021). In the field of powered and intelligent lower limb prostheses, deep learning techniques are being introduced to restore the ambulatory functionality of amputees by providing a human-machine coordination method (Rai et al., 2019; Lee et al., 2021). In earlier research efforts, the conventional phased-based gait assistance approach only aims to produce some level of drive power or a fixed position/torque reference for portable lower limb protheses based on the appearance of distinctive gait events or device states such as heel strike, toe-off or reaching a thigh angle threshold (Tucker et al., 2015; Yu et al., 2016; Lee et al., 2020). This pre-defined finite state action pattern is often discrete, and a series of parameters and thresholds are required to be tuned and fixed, from where the intra-subject, inter-subject differences and unstructured terrains hinder the adaptability and interactive ability of unintelligent prosthetic devices (Au et al., 2008; Liu et al., 2014). Such a control approach is often limited to a specific activity mode, is difficult to personalise and may not fully exploit the capability of the device actuators (Ferreira et al., 2014).

Using deep learning for biometric gait estimation, i.e., limb trajectory estimation based on signals from other correlated limb joints/segments, brings new insights into seamless and personalised trajectory generation for the control of more intelligent devices for a variety of users (Ferreira et al., 2014; Tucker et al., 2015). In contrast to hand-crafted fixed trajectories, this data-driven strategy manages to better trigger terrain-appropriate and biomimetic response through learning the movement pattern from a gait database. Also, the issues involved in prosthetic device adjusting caused by inter-subject and intra-subject difference will be relieved (Tucker et al., 2015).

One of pioneers of complementary limb motion estimation, Vallery et al. (2008) proposed a linear predictor to explore the inter-joint synchronisation in the case of level-walking scenario, and then the staircase also was considered by Vallery et al. (2011). With increasing application of deep learning, many researchers adopted the Long Short-Term Memory (LSTM) neural network as it is well-suited to analysing time sequential physiological movement. Rai et al. (2019) took daily activities into consideration including flat-ground walking both with and without random stops, and stair climbing using a self-decided pace. They concluded that the LSTM model showed an improved performance over linear regression and also Dense Neural Networks (DNN) for an ankle angle prediction task. Zaroug et al. (2021) investigated the applicability of LSTM for the prediction of angular velocity and linear acceleration in the sagittal plane of thigh, shank and foot segments. Inspired by the Sequence to Sequence (Seq2Seq) model in the Natural Language Process (NLP) field, they adopted a two stage Encoder-Decoder LSTM model (Zaroug et al., 2020). Other research has shown that Bidirectional LSTM (Bi-LSTM) provides a modest accuracy increase for human gait cycle judgement (Lee et al., 2021; Zaroug et al., 2021; Hong et al., 2022).

To better capture the time characteristics of highly periodic gait signals, an attention mechanism can be used to evaluate the importance of the timestep, or the input features involved in the LSTM layer. For the temporal attention, more crucial time points in the time sequence will be weighted by higher attention score that can be calculated mathematically (Bahdanau et al., 2014), although attention mechanism also can be used to value the input features rather than timesteps (Qin et al., 2017). Owing to the rare application of the attention mechanism method in the domain of gait kinematics estimation, many researchers cite works from other fields such as NLP or stock price forecasting. Rai et al. (2020) and Wang et al. (2021) adopted an autoregressive attention model developed for NASDAQ 100 Index forecasting (Qin et al., 2017), whereby the calculation of attention score is equivalent to the encoder-decoder attention model used for machine translation in NLP. Specifically, the current hidden state in the decoder queries the hidden state sequence in the encoder and judges its importance when the decoder is outputting a sequence of results (Bahdanau et al., 2014). However, Rai et al. (2020) and Wang et al. (2021) used the motion history of target limb as model inputs to make future prediction for the same target limb, which is not applicable for amputees. Li et al. (2021) and Zhu et al. (2021) weighted input features through adding an attention layer at the beginning before passing them into the LSTM layer, but do not provide a detailed description of the implementation of their feature attention mechanism.

The proposed methods in the literature constitute initial studies into applying an attention mechanism to estimate human motion data, nonetheless the functioning of the attention mechanism needs to be investigated further and the model architecture can be improved. This paper describes a detailed investigation into applying deep learning with an attention mechanism for a continuous biomechanical kinematics estimation task.

## 2 Materials and methods

### 2.1 Data preparation

This study was conducted using a public human locomotion dataset (Sherratt, 2020) collected by the authors’ group from able-bodied participants, where six walking modes were measured, comprising stair ascent/descent, ramp ascent/descent, stopped, level-ground walking and transition between modes were measured (Sherratt et al., 2021). Five 9-axis inertial measurement unit (IMU) sensors consisted of accelerometer, gyroscope and magnetometer are placed in the body locations, two on the legs at the ankles (ankle for short), two on the torso at the hips (hip for short) and one on the chest. More detailed IMU placement on the body can be viewed from previous work of Sherratt et al. (2021). For each IMU sensor, the linear acceleration, the angular velocity, the magnetic field in three axes were measured, and the resultant acceleration of three linear accelerations of triaxial accelerometer was computed by vector sum. The gait database composed of twenty-one subjects was used in our study. The sampling frequency was 100 Hz, and 1.25 million samples were used corresponding to 209 min of data and 10,440 strides in total.

Mapping rules between the physical quantities measured by the IMU signals will be determined using a deep learning model. The resultant linear accelerations of left ankle and left hip were used as the model target outputs, while the IMU signals from the chest, right ankle and right hip as model inputs. Concretely, the measured signals of triaxial accelerator, triaxial gyroscope, triaxial magnetometer and the resultant linear accelerations from three body locations are fed into the model (ten quantities for each IMU and thirty inputs in total). Such a model could be used to control a left leg prosthesis to emulate fully able-bodied movement. Before the start of model training, it is sensible to eliminate noise and outliers by smoothing the measured target output, which is favorable to prevent the neural network from learning inaccurate ground truth. A 5-point Moving Average filter was utilized to smooth the target output.

**FIGURE 1:**
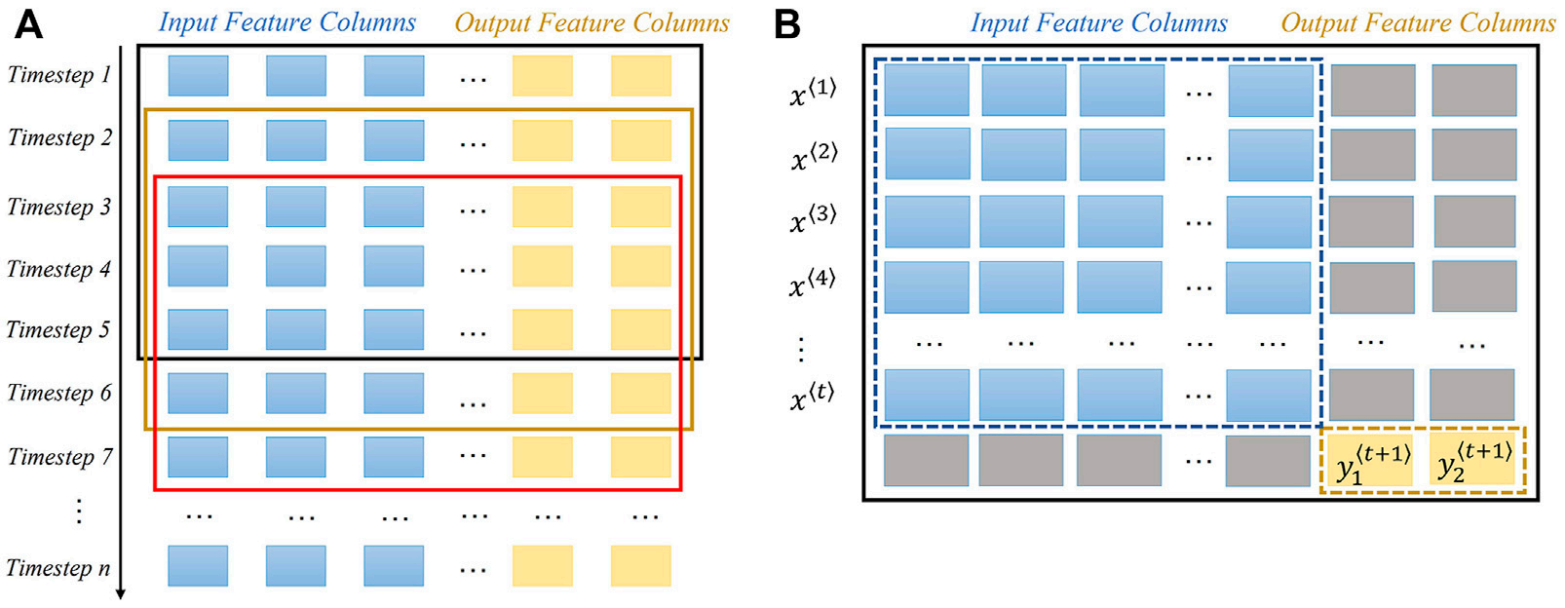
Sliding window illustration for data point resample. There are thirty input features and two output features in total. **(A)** Shows the window sliding process in the sample matrix. Each row represents a timestep, each blue column means an input feature, and each yellow column indicates a target feature. **(B)** Demonstrates the previous t timesteps are formed as one data point to predict two target variables in the next timestep. Blocks coloured grey are the abandoned values.

The tabular data can be viewed as a sample matrix in Figure1A, in which each row represents one timestep and each column contains an input/output feature. To feed time series data points into the standard deep learning pipeline, the original input data should be reshaped as a three-dimensional tensor/array depicted by the shape (No. of data points, No. of timesteps, No. of input features). In the resampling process, a sliding window with fixed length scans the list of samples starting at the beginning of sample matrix, rolling by a step of one timestep until the rolling time window hits the end of sample matrix. For a single rolling window in Figure1B, the input timestep $x^{\langle t\rangle }=\left(x_{1}^{\langle t\rangle },x_{2}^{\langle t\rangle },\cdots ,x_{30}^{\langle t\rangle }\right)$ represents thirty input features at the timestep *t*. A group of shaped data points was formed, by which one single data point contains several input timesteps $x=\left(x^{\langle 1\rangle },x^{\langle 2\rangle },\cdots ,x^{\langle t\rangle }\right)$ and two relevant target features $y=\left(y_{1}^{\langle t+1\rangle },y_{2}^{\langle t+1\rangle }\right)$ in the next timestep. The sequence length in a sliding window is a hyperparameter that needs to be tuned properly during training process.

As for the dataset split, the resampled time-varying history of eighteen individuals were split into a training dataset (85% of the samples) and a cross-validation dataset (15% of the samples), and three unseen participants were left as the testing dataset. The purpose of having a cross-validation dataset was to help adjust hyperparameters, including learning rate and the number of layers. The testing dataset only includes the participants who have been left out of the training and cross-validation datasets. Furthermore, the dataset includes gait data for activity transitions to reproduce a more natural dynamic response (Li and Hsiao-Wecksler, 2013). By ensuring a separate testing dataset the generalization ability of model can be better evaluated, an issue observed in some studies (Liu et al., 2017; Li et al., 2021; Zhu et al., 2021) where the cross-validation dataset was used for testing.

During training, the order of the constructed data points from the sliding window within the training dataset is randomly shuffled to increase robustness. Data normalization is also employed to equally scale the different input features, which is conducive to a fast convergence. The mean value and standard deviation calculated from the training dataset are used for normalization of all the data, including the cross-validation dataset and test dataset for the purpose of same transformation.

### 2.2 Long short-term memory neural network

**FIGURE 2:**
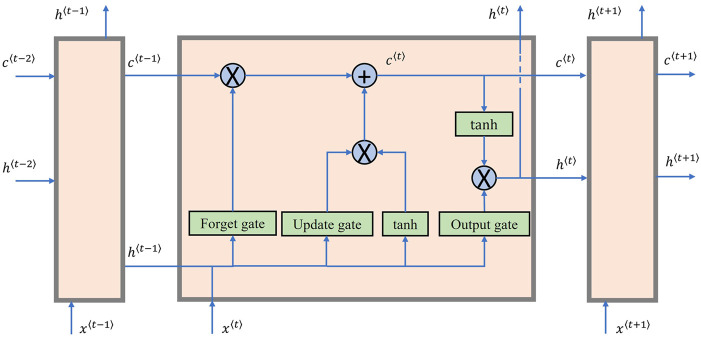
Unfolded LSTM layer showing internal operation for time series.

As an important extension of Recurrent Neural Networks (RNN), LSTM stands out for its ability to reveal temporal sequential dynamics. Time series data $\left(x^{\langle 1\rangle },x^{\langle 2\rangle },\cdots ,x^{\langle t\rangle }\right)$ are processed sequentially and the temporal dependency is revealed through remembering previous informative inputs and forgetting unhelpful ones (Rai et al., 2019). Within the LSTM cell, three gates—the forget gate, update gate and output gate—are introduced to control the internal information flow as shown in Figure 2. In mathematical term, every gate is a sigmoid function whose outputs range from zero to one to decide the forgetting, updating, and output ratio respectively. The hidden state $h^{\langle t\rangle }$ of the *t* ^*th*^ timestep focused more attention on closer timesteps, which endangers the information preservation of the initial timesteps. To alleviate this problem, the memory cell $c^{\langle t\rangle }$ acts as a conveyor belt to retain and deliver signals even if from distant previous timesteps (Lee et al., 2021). From Figure 2, only simple mathematical operations are performed to update the memory cell in each LSTM cell. The LSTM layers can be stacked to have the means for finely extracting intermediate features and finding temporal patterns from low-level input features (Zaroug et al., 2021).

### 2.3 Branched deep learning model

**FIGURE 3:**
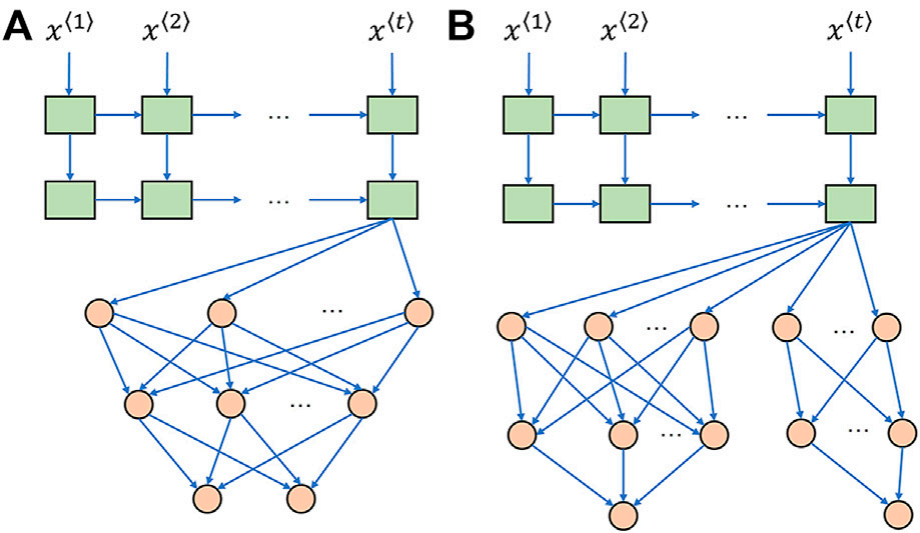
Stacked LSTM network followed by fully connected layers. **(A)** Unbranched baseline model. **(B)** Branched baseline model. LSTM layers and fully connected layers are colored by green and orange, respectively.

As show in Figure 3A, an unbranched structure has no separate hidden layers for a multi-output model, and only the last layer specializes the neural network weights for different output targets. In contrast, the proposed branched model structure in Figure 3B allows information sharing in a few top layers, and the bottom branches retain the specification and characterization function for each individual output target. An experiment was designed to verify if a distraction may occur in the unbranched model topology, although more experiments are required to verify this hypothesis in the future.

### 2.4 Attention model

**FIGURE 4:**
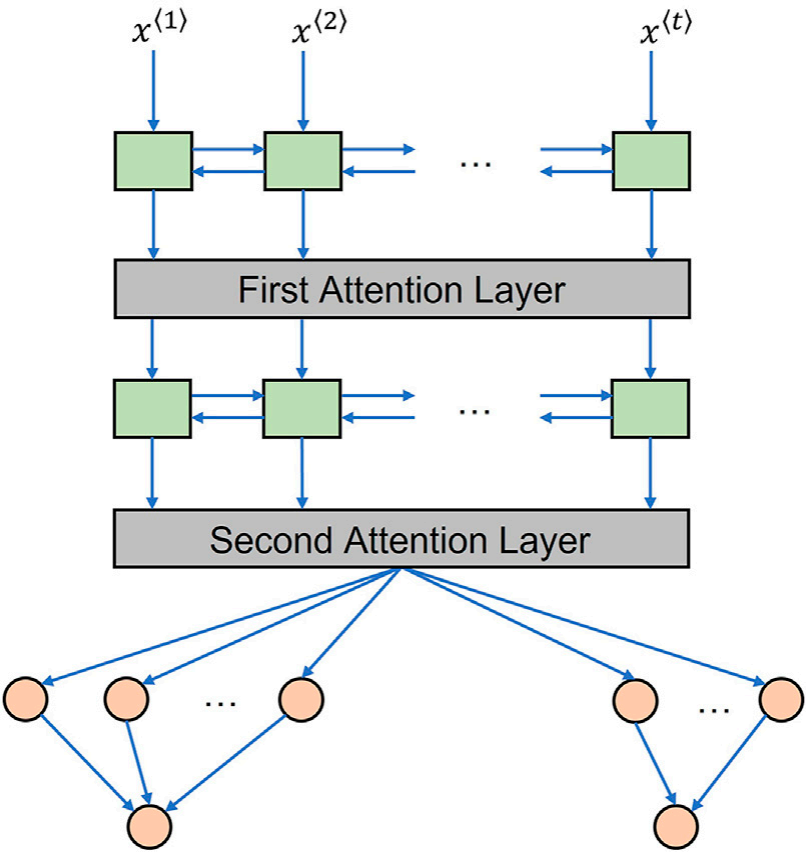
Attention deep learning model with Bi-LSTM.

The attention mechanism is better for processing long time sequences than an LSTM layer alone as it weighs more important timesteps (Rai et al., 2020). This study integrated a concise “feed-forward” temporal attention mechanism that judges the importance of each timestep by multiplying with a corresponding attention score, which can better boost temporal model performance than only using LSTM (Raffel and Ellis, 2015). As a popular combination, the attention model presented in this paper adopts bidirectional LSTM layers. The proposed attention model is shown in Figure 4, in which two similar but slightly different attention layers are inserted downstream of the first and second Bi-LSTM layers.

**FIGURE 5:**
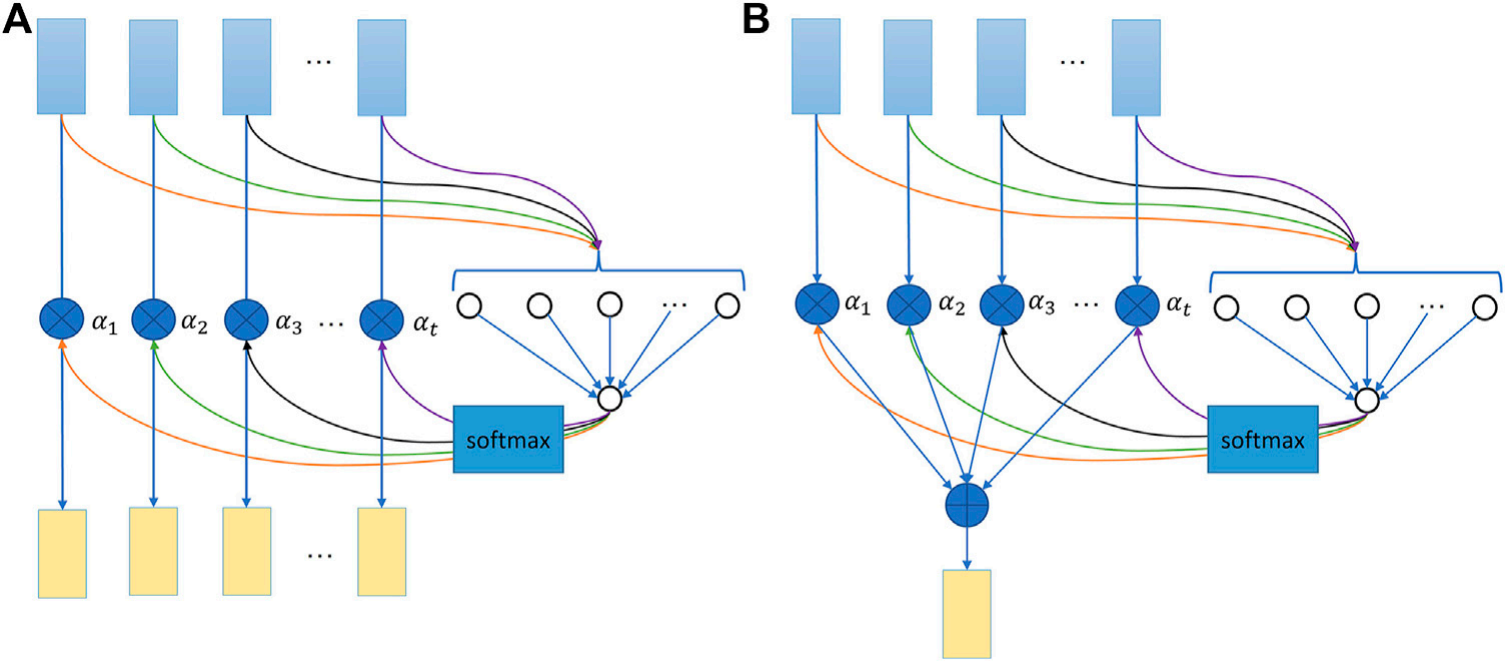
Attention mechanism illustration. **(A)** First attention layer. **(B)** Second attention layer. The attention score calculation for each timestep is independent but using the same learnable function.

For the first attention layer depicted by Figure 5A, each timestep vector output by the first Bi-LSTM layer will be fed into an additional learnable-function inherently multilayer perceptron (MLP). Herein, the specific learnable network weights each timestep based on their input vector features and decides the corresponding attention score timestep by timestep. Then, the learned score results of all timesteps will pass through a SoftMax function to produce normalized the calculated attention scores ( $\alpha_{1},\alpha_{2},\cdots ,\alpha_{t}$ ) (Raffel and Ellis, 2015). The value of each scaled attention score is between zero and one and the summation of all scaled attention scores is one. The second attention layer applies similar working principles as the first attention layer when valuing each timestep from the second Bi-LSTM output sequence. But these weighted timestep vectors are summed up as a single synthetical context vector to properly fit in the following fully connected layers as shown in Figure 5B.

### 2.5 Model training

The deep learning framework TensorFlow (version 2.7.0) was used to train the proposed models, and a single NVIDIA RTX 3060 GPU (8 GB) was deployed to accelerate the training process. An Adaptive Moment Estimation (Adam) optimizer based on gradient descent was used to update the model parameters (Kingma and Ba, 2014). Several hyperparameters, including network size, were determined by a sparse grid search. The timestep length of input window was assigned as 15 and 25 for the unbranched/branched baseline models and the attention model, respectively. The initial learning rate was set as 0.001, and this value is reduced through the learning rate decay technique when the performance improvement for the cross-validation dataset becomes too small. Because training with a larger learning rate is an advantage to quickly approach the optimum solution at the beginning, and then a smaller learning rate is preferred to avoid jumping over and oscillating around the optimum point when the error is small. An early stopping technique also was also used to prevent the occurrence of the overfitting problem.

After a trial of different cost functions, the Mean Absolute Percentage Error (MAPE) was selected as the model loss function, and this metric is used to quantify the model error. MAPE is defined as:

$$MAPE=\frac{1}{n}\overset{n}{\underset{j=1}{\sum }}\left|\frac{y_{j}-{\hat{y}}_{j}}{y_{j}}\right|$$

where $y_{j}$ is the ground truth of *j* ^*th*^ data point, ${\hat{y}}_{j}$ is the prediction value of *j* ^*th*^ data point, and *n* is the total number of data points.

MAPE is a strict measurement method, and the error will be amplified, and its characteristic may contribute to the training process to a certain degree. One classical counterpart is that Glorot and Bengio (2010) adopted the Cross-Entropy loss function instead of Mean Square Error to handle a classification problem, and the amplified loss yielded larger loss function gradients so as to approach the minimum point faster. Two other metrics, Mean Absolute Error (MAE) and Normalized Root Mean Square Error (NRMSE), are also used to assess the results. The NRMSE value, which has been receiving widespread attention recently, quantifies the model error in normalized form (Ardestani et al., 2014; Giarmatzis et al., 2020; Zaroug et al., 2021). NRMSE is defined as:

$$NRMSE=\frac{\sqrt{\frac{1}{n}\sum_{j=1}^{n}{\left(y_{j}-{\hat{y}}_{j}\right)}^{2}}}{\max\left(y\right)-\min\left(y\right)}$$

where *y* is the ground truth of entire dataset. MAE is defined as:

$$MAE=\frac{1}{n}\overset{n}{\underset{j=1}{\sum }}\left|y_{j}-{\hat{y}}_{j}\right|$$

For the branched model structure, each output (ankle/hip) has its own loss function. But unbranched layout only has one overall loss function that simply takes the average of the loss of each output. Moreover, the previously mentioned MLP learnable function in the attention mechanism is a part of the whole attention model, therefore no extra cost function is required for this additional component.

## 3 Results

### 3.1 Bi-LSTM for attention model

As previously stated, Bi-LSTM layers instead of LSTM layers were selected for the attention model in this study. The justification for this choice is shown in Table 1, where Bi-LSTM outweighs LSTM for ankle and hip prediction in all three metrics. For the branched baseline model, neither LSTM nor Bi-LSTM showed a significant advantage. These models were all well-trained initially using both the training dataset and cross-validation dataset. The results presented are the errors in resultant acceleration predictions at ankle and hip for the testing dataset.

**TABLE 1:** The Comparison of Model Performance with LSTM/Bi-LSTM using testing data set.

Metric	Architecture	Ankle	Hip
NRMSE	**Branched Baseline with LSTM**	10.44%	7.93%
	Branched Baseline with Bi-LSTM	9.97%	8.04%
	Branched Attention with LSTM	10.48%	7.98%
	**Branched Attention with Bi-LSTM**	9.06%	7.64%
MAE (*m/s* ^*2*^)	**Branched Baseline with LSTM**	3.47	1.51
	Branched Baseline with Bi-LSTM	3.53	1.49
	Branched Attention with LSTM	3.63	1.53
	**Branched Attention with Bi-LSTM**	3.20	1.43
MAPE	**Branched Baseline with LSTM**	23.82%	14.01%
	Branched Baseline with Bi-LSTM	24.85%	13.66%
	Branched Attention with LSTM	23.91%	13.86%
	**Branched Attention with Bi-LSTM**	21.99%	13.37%

The selected models are **bold**.

### 3.2 Estimation error and plots

The four models chosen, i.e., the unbranched smaller/larger baseline models and the branched baseline/attention models, were all well-trained on both of training dataset and cross-validation dataset. They were then evaluated on the testing dataset regarding two aspects, the metric error and model robustness. The three metrics, NRMSE, MAE, and MAPE, were used to assess model accuracy.

All four models have achieved similar errors on the training dataset and cross-validation dataset, providing confidence that no overfitting has occurred during training stage. The NRMSE training errors for resultant ankle and hip acceleration prediction are around 2.6% and 2.2% respectively, and the cross-validation errors are about 3.1% and 2.7% respectively. Table 2 compares the model performance for the three test subjects who are excluded from the model training process; their gait data have not been included in either the training data used to update model parameters, or the cross-validation data used to tune model hyperparameters. By comparing the smaller/larger unbranched baseline model, simply increasing the network size cannot improve model testing performance. A slight improvement can be seen after switching the model structure to the branched baseline model. The attention model broke through a bottleneck and produced consistently superior testing results than the three baseline models with regard to all metrics and this phenomenon is more significant for the ankle motion.

**TABLE 2:** Model evaluation on the testing dataset (error in resultant acceleration predictions).

Metric	Architecture	Ankle	Hip
NRMSE	Unbranched Smaller Baseline	10.54%	7.69%
	Unbranched Larger Baseline	11.83%	7.84%
	Branched Baseline	10.44%	7.93%
	Branched Attention	**9.06%**	**7.64%**
MAE (*m/s* ^*2*^)	Unbranched Smaller Baseline	3.69	1.47
	Unbranched Larger Baseline	3.95	1.48
	Branched Baseline	3.47	1.51
	Branched Attention	**3.20**	**1.43**
MAPE	Unbranched Smaller Baseline	25.19%	14.12%
	Unbranched Larger Baseline	25.83%	13.83%
	Branched Baseline	23.82%	14.01%
	Branched Attention	**21.99%**	**13.37%**

Values in **bold** represent the best result on the testing dataset for each metric.

**FIGURE 6:**
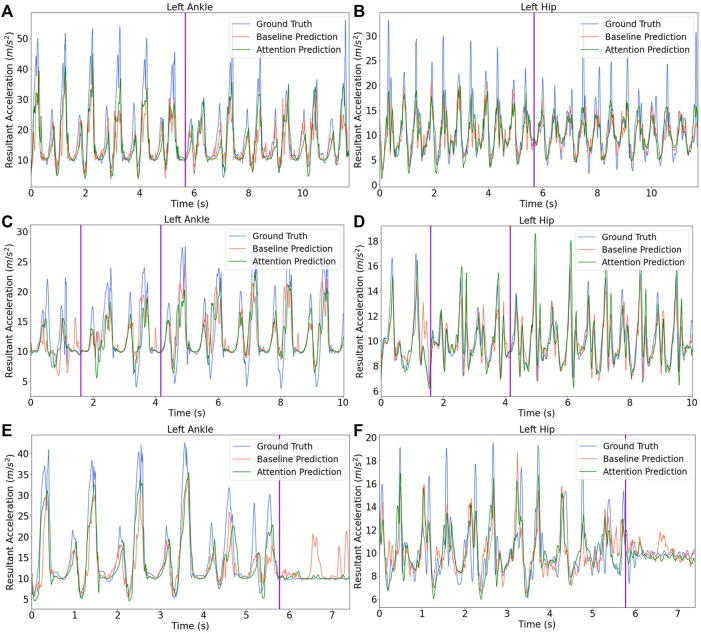
Model prediction on testing data across varied activities. The activity transitions are marked by purple vertical lines. **(A)** and **(B)** depict the transition from walking uphill to downhill for ankle and hip, respectively. **(C)** and **(D)** first present the transition from walking upstairs to downstairs and then show the switch from stair descent to level-walking for ankle and hip, respectively. **(E)** and **(F)** show the transition from level-walking to stopped for ankle and hip, respectively.

This research also visually compared the estimation outcomes from the branched baseline model and the branched attention model on the testing dataset. The plots in Figure 6 examine the activity transition 1) from uphill to downhill, 2) from stair ascent to stair descent then to level-walking and 3) from level-walking to stopped. From Figures 6A–D, the generated acceleration trajectories during the transition period between two activities were considered as acceptable for both ankle and hip, which reflects the concept of seamless control. It also appears the attention model can capture the trend change more precisely than the branched baseline model. The prediction error mainly comes from the imperfect forecast of amplitude in peaks and the time shift between ground truth signals and corresponding predictions. However, the branched baseline model failed to accommodate a transition from level-walking to stopped, and a few wrongly predicted peaks can be observed in Figures 6E,F.

### 3.3 Model robustness and size analysis

**FIGURE 7:**
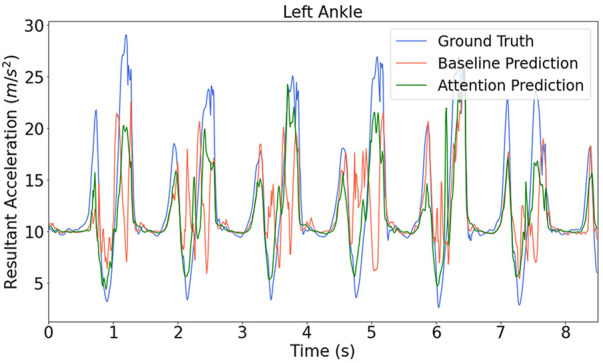
Model prediction for the ankle acceleration on the testing data from up-stairs.

The predictions from the branched baseline model sometimes exhibited much more fluctuation than the attention model, for instance in Figure 7. The dynamic characteristics of the error cannot be shown by error metric values, so the branched attention model and its counterpart branched baseline model are compared in the frequency domain.

**FIGURE 8:**
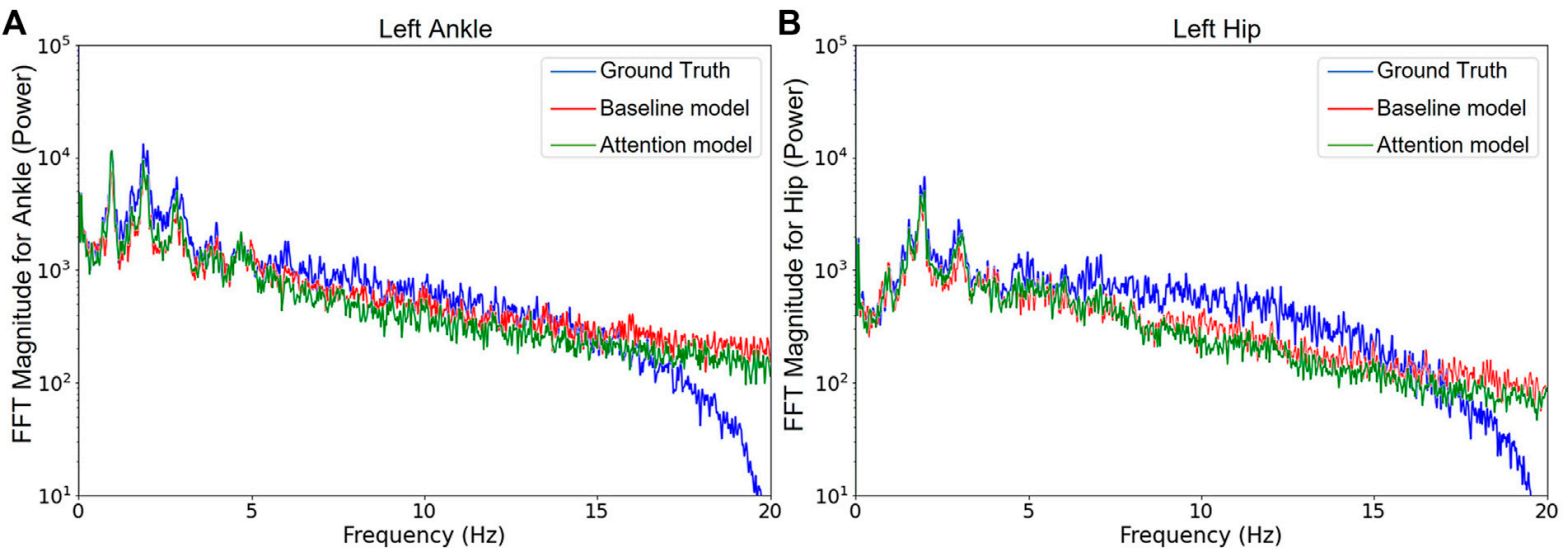
Fast Fourier analysis for the ground truth and the prediction results of models. **(A)** and **(B)** are about left ankle and hip resultant acceleration, respectively. The branched attention model and the branched baseline model are used.

After conducting a Fast Fourier Transform (FFT) on the ankle and hip acceleration prediction results and the ground truth for the test subjects, the difference of power distribution over a range of frequencies for the two paired models is depicted in Figure 8. Compared to the branched baseline model, the attention model prediction was found to slightly exhibit more power in the low-frequency range. In contrast, the baseline model prediction has more power in the high-frequency range than the attention model. The boundary between these ranges is around 6 Hz for both ankle and hip. Commonly for this application, it is agreed that most discriminative components are contained in the frequency range below 6 Hz (Mäkela et al., 2021). For that reason, many researchers now focus on the 0—6 Hz frequency range when pre-processing biomechanical gait data (Luo et al., 2020; Renani et al., 2021). The results indicate that the attention model creates less high frequency variation (or noise) above the frequency range of interest than the baseline model. However, the amplitudes of baseline and attention models are both lower than the ground truth in the low frequency range, and their power do not decay as fast as the ground truth after 15 Hz. Usually, very few signals above 15 Hz are beneficial to describe human movement (Rai et al., 2020).

Compared to the branched baseline model, the network size of the attention model was scaled down by 57% in term of model parameters (308362 versus 132292 parameters). We created a new model in accordance with the branched attention model architecture but removing the two attention layers. With this model, NRMSE training errors less than 8.15% and 7.88% for ankle and hip respectively could not be achieved, and testing errors were 14.81% and 12.09% respectively. The outcome without attention layers became much worse than the model with attention mechanism (9.06% and 7.64% testing error for ankle and hip respectively).

Additionally, the influence of filtered inputs has been investigated based on the proposed attention model architecture. The MAPE errors are 22.09% (ankle) and 12.98% (hip) for the filtered inputs by the 5-point Moving Averaging filter; the MAPE errors are 22.99% (ankle) and 12.36% (hip) for a 2nd order Butterworth filter with 6 Hz cut-off frequency. With our 100 Hz sampling frequency, the Moving Average filter is less aggressive, which means that it is less likely to remove useful parts of the signals although more noise will remain. The Butterworth filter gives a small advantage for the hip prediction but makes the ankle prediction worse in this case.

## 4 Discussion

### 4.1 Main findings

From the comparison of the unbranched smaller/larger baseline models it appears that purely increasing the size of unbranched model does not offer benefits in term of error reduction. The unbranched larger baseline model nearly doubled the smaller model in total number of model parameters. This issue is due to the limited potential of the model structure, and simply increasing network size cannot bring extra benefits in such an already large enough neural network (He et al., 2016). To tackle this challenge, the branched model architecture was adopted to better specialize the regression prediction for the multioutput biomechanics problem notably for joints/segments with a larger range of movement (e.g., ankle), and revealed modest improvement in MAE and MAPE metrics but not NRMSE. A great deal of effort has been expended to search for the optimum hyperparameters (including network size) to gain a clear advantage for the branched baseline model, and more datasets and tests are necessary to confirm architectural validity in the future. The attention model leveraged the prediction performance further and possessed a distinct advantage particularly for ankle kinematic trajectory generation, and less time was consumed to find out a feasible solution for hyperparameters and model size. Aside from error reduction, the attention mechanism significantly decreased the neural network size as more attention is devoted to more crucial timesteps in the time series. Rai et al. (2020) and Bahdanau et al. (2014) have illustrated and visualized the mechanism of allocating attention. In our work, an experiment was conducted to demonstrate that the attention mechanism has potential to reduce model size while improving model performance. The evidence indicates that a small model without attention is inclined to be underfitted thus barely extracting enough information from the training data. The FFT analysis results confirmed that the attention mechanism focused the model fit to the meaningful low frequency range of the signal spectrum.

Many intelligent prosthesis and exoskeleton research practitioners (Liu et al., 2017; Lee et al., 2021; Zaroug et al., 2021) concentrated on simple movement scenarios such as walking on a treadmill or level-ground and often at constant speeds. In contrast, our study included slopes and stairs and random transitions between two consecutive walking activities. Although some high-intensity activities (e.g., stair ascent navigation) are a challenge to estimate, the experiments proved that the presented mode-free trajectory generator has potential to adapt to an unstructured environment and varied legged locomotion tasks. Due to the shortage of relevant theories in the field of biomechanical parameter prediction, some researchers (Rai et al., 2020; Wang et al., 2021) were compelled to mechanically apply an existing attention model (Qin et al., 2017). The downside is that their models took in the historical gait data of the target leg from previous timesteps to predict the future trajectory for the same leg. Clearly, it is not possible to acquire an amputee’s historical gait data from the missing target limb as model inputs in practice. For a subject with locomotive impairments, the orchestrated movement profile should be inferred from his/her remaining healthy limbs instead of the affected limb itself.

The concept of end-to-end deep learning was illustrated in this study as the raw input features were not filtered or deliberately selected, and all of them were fed into the training process directly to verify if the neural network can extract principal signals from original lower-level IMU data samples. Nonetheless, the abnormal outliers and high frequency noise existing in the target signals on the dataset should be eliminated by a filter before using them to update model parameters, as these imperfect characteristics of the original target features are not expected to be learned by the model. The selection of input features also is a time-consuming process, and some components of the IMU signals might be found to make little contribution to the prediction precision (Renani et al., 2021). Before the rise of deep learning, some feature engineering methods such as Principal Component Analysis (PCA) remain popular to pre-processing gait data, and then the transformed data were inputted into a regressor/classifier based on a traditional machine learning algorithm such as a Support Vector Machine (SVM) or Decision Tree (DT) (Boe et al., 2021; Lotfi and Kedir-Talha, 2022). However, all measurable IMU physical quantities were directly fed into the neural network models in this work, and we rely on the automatic extraction of beneficial intermediate features and abandonment of useless components.

### 4.2 Limitations

Regardless of model topology, flaws in data quality also diminished the maximum effectiveness of the model architecture, since the data samples of each subject collected by Sherratt et al. (2021) are moderately uneven in terms of activity type and duration time. All the participants were permitted to perform their behaviors in a voluntary way with a self-selected pace and without a fixed routine, leading to unbalanced sample problems between subjects and between activities. Besides, their dataset does not collect the motion of knees. Traditional theoretical research into machine learning pays attention to the model-centric method based on standard datasets, but a practical industrial application should give higher priority to data-centric methods whereby the quality of data should be valued more (Strickland and Ng, 2022; Zhong et al., 2022). For the purpose of designing a novel model architecture rather than devising a commercial product, the accelerations of body parts are selected as model outputs, more commonly-used control signals such as limb joint angles should be investigated to further validate the generalizability and effectiveness of our proposed model.

## 5 Conclusion

State-of-the-art deep learning topologies were investigated for estimating walking gait movements, and an attention mechanism was shown to improve the performance of a recurrent neural network to give better prediction accuracy and smaller network size. The movement predictor receives as inputs complementary limb motion. It can handle slopes and stairs and achieve seamless prediction during transition between varied walking activities. Consequently, this allows continuous control without the need to separately identify each activity and delineate each phase event during a gait cycle. This contrasts with the discontinuous finite-state machine approach that demands high technical expertise to set up hand-crafted rules and pre-defined fixed trajectory references. Although the flexible trajectory planning with deep learning techniques for biomechatronic prostheses is becoming a much-researched topic, it might be more practical to implement this concept in more tractable and safer rehabilitation devices such as exoskeletons. Furthermore, the deep learning-driven modelling approach also promises to allow estimation of the hard-to-measure joint kinematic parameters (e.g., joint contact force/moment), as an alternative to building up complex musculoskeletal models to allow estimation from simulations driven by motion capture data.

Normally, a data-driven model needs to consume a large and high-quality dataset to make certain of personalization and generalization. Additional datasets should be identified or collected to validate the proposed models further. Considering the high expense of collecting locomotion datasets in terms of time, funds and personal privacy, future work will place great expectations on the data augmentation techniques such as Generate Adversarial Networks (GAN) in order to better train machine learning models with fewer available data. Other attention mechanisms such as self-attention also are worth being investigated. The proposed attention model structure is not only confined to this study but can provide a useful reference for other types of multi-input and multi-output estimation tasks.

## Data Availability

Publicly available datasets were analyzed in this study. This data can be found here: https://doi.org/10.5281/zenodo.4390499.
